# Impact of Microstructure on Sensing Performance of Fiber-Based Iontronic Pressure Sensors: A Comparative Study

**DOI:** 10.3390/s25216711

**Published:** 2025-11-03

**Authors:** Cheng Liu, Jiaxin Xu, Shiman Yang, Yihan Xu, Jianyu Wang, Xiaoqing Liu, Li Wang, Yichun Ding

**Affiliations:** 1 School of Physics and Materials Science, Nanchang University, Nanchang 330031, China; chengliu02@163.com (C.L.); xujiaxin@fjirsm.ac.cn (J.X.); shimanyang1@163.com (S.Y.); 5714123050@email.ncu.edu.cn (Y.X.); jywang@ncu.edu.cn (J.W.); liwang@ncu.edu.cn (L.W.); 2 Fujian Institute of Research on the Structure of Matter, Chinese Academy of Sciences, Fuzhou 350002, China; 3 Jiangxi Provincial Key Laboratory of Photodetectors, Nanchang University, Nanchang 330031, China; 4 Research Center for Chip Design, Nanchang University, Nanchang 330031, China

**Keywords:** iontronic pressure sensors, ionic liquids, fiber materials, porosity, microstructure, wearable sensing

## Abstract

Highly sensitive flexible pressure sensors are crucial for wearable health monitoring and human–machine interaction. While the emerging iontronic sensors inherently offer high sensitivity, this can be further improved by engineering microstructured interfaces. In this study, we employ four different types of common fiber materials as substrates for fabricating ionic dielectric layers by a simple impregnation of ionic liquid (IL). A comparative study reveals that the porosity and microstructural architecture (e.g., fiber diameter) of the substrate material directly influences the amount of adsorbed IL and consequent sensing performance. We achieved the highest sensitivity by using a thin electrospun TPU/IL nanofiber mat (33 μm), which exhibited high sensitivities of 3.10 kPa^−1^, 1.85 kPa^−1^, and 1.02 kPa^−1^ in the pressure ranges of 0–200 kPa, 200–400 kPa, and 400–700 kPa, respectively. Furthermore, the sensor exhibited an excellent fast response (2.71 ms) and recovery time (8.71 ms), along with outstanding cyclic stability. This work provides valuable guidance for selecting and utilizing common fiber materials to develop high-sensitivity iontronic pressure sensors, paving the way for their practical application in next-generation wearable electronics.

## 1. Introduction

With the rapid advancement of embodied artificial intelligence, robotics, and the Internet of Things, the demand for tactile sensing technology has grown dramatically. Pressure sensors, as key components for detecting external force, are widely used in numerous fields [1,2,3,4,5,6]. However, the mainstream pressure sensors based on conventional silicon-based materials and microfabrication techniques can hardly withstand large deformations and conform to curved surfaces due to their inherent rigid characteristics [7,8]. This not only causes signal inaccuracies but also greatly limits their application ranges. In contrast, flexible pressure sensors utilizing polymer substrates such as polydimethylsiloxane (PDMS) and Ecoflex [9,10] offer excellent flexibility, stretchability, and lightweight properties. These properties are essential for emerging applications in robotic tactile sensing, electronic skin, and wearable health monitoring [11,12,13,14].

Pressure sensors generally operate based on four mechanisms: piezoresistive, piezoelectric, triboelectric, and capacitive [15,16,17,18]. Among them, capacitive pressure sensors are widely used due to their advantages of low power consumption, both static and dynamic pressure monitoring capability, and simple structure [19]. Conventional capacitive pressure sensors are constructed by sandwiching a dielectric layer between two electrodes. According to the equation of capacitance, *C* = *εA*/*d* (where *C*, *ε*, *A*, and *d* is capacitance, dielectric constant, electrode contact area, and thickness of dielectric layer, respectively), researchers optimized the sensing performance of capacitive pressure sensors typically by manipulating the three key variables: (1) by modulating the dielectric constant of dielectric layer by the introduction of conductive fillers such as carbon nanotubes [20,21] and silver nanowires [22,23]; (2) by optimizing the compressive deformation through the use of a dielectric layer with low elastic modulus [24]; and (3) by constructing microstructured interfaces such as pyramids [25,26,27], porous hierarchies [28,29], and micropillar arrays [30,31] on the surface of dielectric layer. While these strategies can significantly enhance performance, the sensitivity of dielectric-based capacitive pressure sensors often remains insufficient for detecting subtle physiological signals, which is critical for health monitoring applications.

Recently, a new type of capacitive pressure sensor, termed as iontronic capacitive pressure sensor, was developed based on an ionic conduction mechanism [32,33]. Unlike conventional capacitive pressure sensors that rely on electronic conduction, iontronic pressure sensors utilize ion migration to form an electric double layer (EDL) at the electrode/electrolyte interface. This EDL mechanism generates extremely high capacitance, enabling ultra-high sensitivity. The iontronic pressure sensors are typically fabricated by replacing the conventional dielectric layer with an ionic material, such as a film incorporated with ionic liquid (IL) or a polyelectrolyte film. ILs are usually encapsulated within a polymer/gel matrix or absorbed into a porous substrate. Based on the EDL mechanism, increasing the concentration of IL can effectively enhance the capacitance of the interfacial bilayer. However, an excess of IL can lead to negative effects, including leakage [34,35] and a significant increase in the gel viscosity [36]. Similarly to dielectric-based capacitive sensors, the sensitivity of iontronic pressure sensors can also be significantly enhanced by constructing microstructures to regulate the interfacial contact area. For example, Niu et al. [37] constructed pyramidal microarrays on the surface of an ionic layer and achieved a sensitivity of up to 655.3 kPa^−1^. Other microstructures include sandpaper textures [38,39,40], multistage microcolumns [41], pleats [42], and interlocks [43], all of which have been shown to greatly improve sensing performance. In particular, Guo’s team [44] developed a reduced-order inverse design framework that theoretically simulates the required microstructure parameters to meet a target performance, offering a powerful tool for the on-demand customization of sensors. However, producing these microstructures typically requires complex and costly processes like photolithography, multi-step transfer printing, or high-precision 3D printing [45,46], which hinder their potential for mass production.

Fiber materials [47,48] such as nonwovens and textiles are ideal for making iontronic pressure sensors due to their innate porous structure that can load significant amounts of IL, serving as an effective ionic layer. In addition, the inherent microstructures of their constituent fibers can significantly enhance the sensitivity. Furthermore, their natural porosity enables the development of permeable electronics to enhance the comfortability. In this study, we present a comparative analysis of four types of fiber materials, including (a) woven fabric, (b) cotton nonwoven fabric, (c) viscose spunlace fabric, and (d) electrospun nanofiber mats, for fabricating iontronic pressure sensors by impregnation of IL. Their distinct structural properties (e.g., fiber diameter, arrangement, porosity, and thickness) led to significant differences in IL absorption and subsequent sensing performance. Results demonstrate that the electrospun nanofiber mat, characterized by small fiber diameter, high porosity, and low thickness, synergistically enhanced both IL loading capacity and reversible deformation. This resulted in a sensor with exceptional sensitivity, stability, and a wide detection range. The optimized sensor achieved high sensitivities of 3.10, 1.85, and 1.02 kPa^−1^ within the 0–200, 200–400, and 400–700 kPa ranges, respectively, alongside rapid response and recovery times of 2.71 ms and 8.13 ms. Owing to this comprehensive performance, the sensor was successfully deployed for wearable physiological monitoring, accurately capturing diverse physiological signals. This provides a reliable technological platform for applications in health monitoring and human–machine interaction.

## 2. Materials and Methods

*Materials*: Ionic liquid (1-ethyl-3-methylimidazolium bis(trifluoromethyl sulfonyl) imide ([EMIM][TFSI])) (99%) was obtained from Shanghai Macklin Bio-Chem Technology Co., Ltd., Shanghai, China. Anhydrous ethanol was purchased from Xilong Science Co., Ltd., Shantou, China. Cotton nonwoven fabric was acquired from Suzhou Oude Dust-free Materials Co., Ltd., Suzhou, China. Viscose spunlace fabric (100% viscose) was acquired from Jiangxi Xinwang Sanitary Products Co., Ltd., Shangrao, China. Electrospun thermoplastic polyurethane (TPU) nanofiber mats (33 μm, 43 μm, and 201 μm) were purchased from Jiangxi Xiancai Nanofiber Technology Co., Ltd., Nanchang, China. The woven fabric is sourced from discarded clothing.

*Preparation of fiber-based ionic layer*: The fiber substrates were first cleaned by ultrasonication in anhydrous ethanol for 1 h to remove impurities. Subsequently, they were cut into small pieces, and immersed in a mixture of [EMIM][TFSI]/ethanol (1:9) for 1 h to allow for IL absorption. Finally, the IL-infused fiber materials were dried at 70 °C to evaporate any residual ethanol.

*Fabrication of pressure sensor*: The pressure sensors were constructed in a sandwich architecture. PET/Au films, prepared by successive evaporation of Cr (5 nm) and Au (100 nm) layers onto a 20 μm PET film via thermal evaporation, were used as the flexible electrodes. The fiber-based ionic layer (1 × 1 cm^2^) was sandwiched between two PET/Au electrodes. Silver wires were attached to the electrodes using silver paste to serve as external electrical connections. Finally, the entire assembly was sealed with 3M tape to ensure mechanical stability during testing. The structural diagram of the sensor is shown in Figure S1.

*Characterization and measurement*: The microstructure and elemental composition of the fiber-based ionic layers were characterized using a high-resolution field emission scanning electron microscope (FESEM, ZEISS/GeminiSEM 300, Carl Zeiss AG Co., Ltd., Oberkochen, Germany) equipped with an energy dispersive X-ray spectroscopy (EDS). Fourier-transform infrared (FT-IR) spectroscopy of ionic layer was performed on a Bruker spectrometer (Thermo Nicolet iS5, Bruker Tensor II, Berlin, Germany). The capacitance of the iontronic pressure sensors was measured at 5 kHz using a precision LCR meter (TH2840B, Changzhou Tonghui Electronics Co., Ltd., Changzhou, China). For pressure sensing test, mechanical compression was administered using a computer-controlled stage integrated with a force gauge (XLD-20E, Jingkong Mechanical Testing Co., Ltd., Guangzhou, China). All measurements were conducted on sensors with a size of 7 mm × 7 mm.

## 3. Results and Discussion

### 3.1. Preparation and Characterization of Fiber-Based Ionic Layers

**Figure 1:**
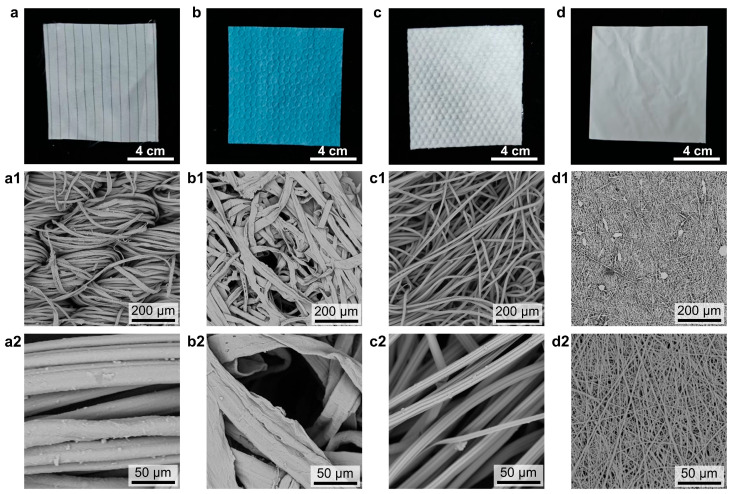
Optical images of the four types of fiber substrates: (**a**) Woven fabric, (**b**) Cotton nonwoven fabric, (**c**) Viscose spunlace fabric, and (**d**) Electrospun TPU nanofiber mat. (**a1**,**a2**) SEM images of woven fabric; (**b1**,**b2**) SEM images of cotton nonwoven fabric; (**c1**,**c2**) SEM images of viscose spunlace fabric; (**d1**,**d2**) SEM images of electrospun TPU nanofiber mat.

We rationally selected four representative fiber-based substrates for preparing the ionic layer of iontronic pressure sensors: woven fabric, nonwoven fabric, spunlace nonwoven fabric, and electrospun nanofiber mat. The thicknesses of woven fabric, cotton nonwoven fabric, and viscose spunlace fabric are 410 μm, 243 μm, and 670 μm (with a semicircular surface thickness of 427 μm), respectively. The first three substrates are composed of conventional natural or synthetic fibers with diameters typically ranging from a few to tens of micrometers, but they are assembled using different techniques: weaving, hot pressing, and hydroentanglement (spunlacing), respectively. In contrast, the electrospun nanofiber mat is a specialized nonwoven material produced by the electrospinning technique, characterized by fiber diameters on the scale of hundreds of nanometers. Figure 1 presents optical and corresponding SEM images of the four selected fiber substrates. As shown in Figure 1a, the woven fabric consists of precisely interwoven twisted yarns, creating a regular macroscopic texture. The cotton nonwoven fabric is composed of randomly overlaid cotton fibers (Figure 1b). Its surface features periodic concave micro-patterns formed by the hot-pressing process, and the individual fibers appear irregular. The viscose spunlace nonwoven fabric is produced by entangling viscose rayon fibers with high-pressure water jets, resulting in a soft, strong, and absorbent material without binders. Its surface shows a regular array of microsphere bumps, with a highly hollow and porous structure (Figure 1c). The electrospun TPU nanofiber mat consists of randomly overlaid nanofibers (Figure 1d). These nanofibers are created by applying a high voltage to a polymer solution jet, which stretches and accelerates the jet to form fine fibers. This nanoscale morphology endows the mat with a high specific surface area and porosity.

**Figure 2:**
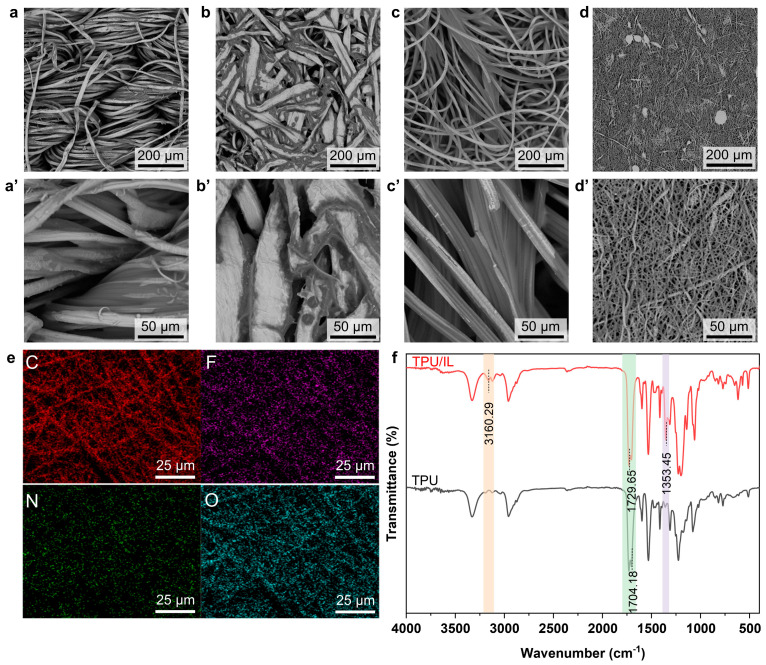
(**a**–**d**) SEM images of the fiber substrate absorbed with IL: (**a**) Woven fabric/IL, (**b**) Cotton nonwoven fabric/IL, (**c**) Viscose spunlace fabric/IL, and (**d**) Electrospun TPU nanofiber mat/IL. (**a′**–**d′**) Zoom-in SEM images of the fiber substrate absorbed with IL: (**a′**) Woven fabric/IL, (**b′**) Cotton nonwoven fabric/IL, (**c′**) Viscose spunlace fabric/IL, and (**d′**) Electrospun TPU nanofiber mat/IL. (**e**) SEM-EDS elemental mapping images of the TPU/IL. (**f**) FT−IR spectra of TPU and TPU/IL.

Owing to the porous structure of the fiber substrates, the ionic layer can be straightforwardly prepared via simple impregnation of IL. This process involved immersing the materials in a mixed [EMIM][TFSI]/ethanol solution followed by drying, resulting in a homogeneous IL coating. As can be seen in the SEM images (Figure 2a–d), an ultrathin IL film can be identified on the fiber surfaces. The IL absorption capacity varied significantly across the different materials (Figure S2). The woven fabric and cotton nonwoven fabric exhibited low IL adsorption (~15%), attributable to the woven fabric’s dense interwoven structure and the nonwoven’s hot-rolled fiber bonding. In contrast, the highly porous viscose spunlace fabric and electrospun nanofiber mat showed substantially higher IL loading. The loading on the TPU nanofiber mat increased as its thickness decreased, with a maximum loading of ~57% achieved on a 33 μm thick mat. This is primarily due to the thinner nanofiber mat providing a shorter diffusion path and higher specific surface area, which synergistically increase the IL loading capacity.

The successful coating of IL was confirmed through energy dispersive X-ray spectroscopy (EDS) and Fourier transform infrared (FT-IR) spectroscopy. For the TPU/IL nanofiber mat, EDS elemental mapping (Figure 2e) detected fluorine (F)—a key element from [TFSI]^−^—in addition to the carbon (C), nitrogen (N), and oxygen (O) inherent to the TPU polymer. The FT-IR spectra provided further evidence: new characteristic peaks emerged at 3160 cm^−1^ and 1353 cm^−1^, corresponding to the C-H stretch on the [EMIM]^+^ imidazole ring and the CF_3_ group in [TFSI]^−^, respectively (Figure 2f). Crucially, the original C=O stretching vibration peaks of TPU at ~1730 cm^−1^ remained unchanged, indicating a stable, non-destructive integration of the IL with the TPU substrate [49,50]. Consistent results confirming successful IL loading were also observed for the other fiber substrates (Figures S3–S6). Importantly, despite the IL impregnation, the highly porous nature of all fiber materials was preserved, granting the composites excellent gas permeability. This was demonstrated by a water evaporation rate equivalent to that of an open container over a five-day period (Figure S7).

### 3.2. Performance of Fiber-Based Iontronic Pressure Sensors

The operating principle of iontronic pressure sensor differs from that of conventional capacitive sensors. While conventional sensors rely on transient electronic polarization, the iontronic pressure sensor leverages the formation of an EDL on the surface of the electrodes. The large capacitive contribution from the EDL constitutes the main source of capacitive signal. Consequently, the total capacitance of the sensor (*C_EDL_*) can be expressed as [51,52]:

$$C_{EDL}={\left(\frac{1}{C_{H}}+\frac{1}{C_{D}}\right)}^{-1}=\eta_{A}\cdot \varphi (d,\varepsilon ,C,\phi ,T)\cdot A=\mathrm{UAC}\cdot A$$

where *C_H_* and *C_D_* represent the capacitance of the bilayer at the interface of the upper and lower electrodes, respectively; *A* denotes the contact area between the ionic layer and the electrode, and *φ* (*d*, *ε*, *C*, *φ*, *T*) is a composite parameter affected by the thickness of the Helmholtz layer (*d*), the dielectric constant (ε), the ionic species and concentration (*C*), the surface potential (*φ*), and the temperature *T*. When pressure is applied, the dielectric layer is compressed, resulting in an increase in its contact area with the electrode, and at the same time, the close contact decreases the effective thickness of the ionic layer, which significantly increases the interfacial capacitance and ultimately causes a change in the total capacitance. The sensor sensitivity (*S*) is defined as *S* = (Δ*C*/*C*_0_)/Δ*P*, where Δ*C* is the capacitance change, *C*_0_ is the initial capacitance, and *P* is the applied pressure. Based on the ion-mediated mechanism, even minimal pressure variations can trigger a substantial capacitive response, resulting in high sensitivity. Additionally, as pressure is applied, the effective permittivity of the dielectric layer also changes. Based on the Maxwell-Garnett equivalent medium theory model, we can derive the *ε_eff_* of the dielectric layer:

$$\frac{\varepsilon_{eff}-\;\varepsilon_{fiber}}{\varepsilon_{eff}+2\varepsilon_{fiber}}=f_{air}\frac{\varepsilon_{air}-\;\varepsilon_{fiber}}{\varepsilon_{air}+2\varepsilon_{fiber}}+f_{IL}\frac{\varepsilon_{IL}-\;\varepsilon_{fiber}}{\varepsilon_{IL}+2\varepsilon_{fiber}}$$

where *ε_eff_*, *ε_fiber_*, *ε_IL_*, and *ε_air_* represent the relative permittivity of the equivalent medium, fiber, interlayer (IL), and air, respectively, and *f_air_* and *f_IL_* denote the volume fractions of air and IL within the dielectric layer [53]. Before pressure application, air dominates, resulting in a relatively low permittivity. Under pressure, air molecules are progressively replaced by ionic liquid, causing *f_IL_* to increase significantly. This leads to a sharp rise in *ε_eff_*, further enhancing the sensor’s sensitivity.

**Figure 3:**
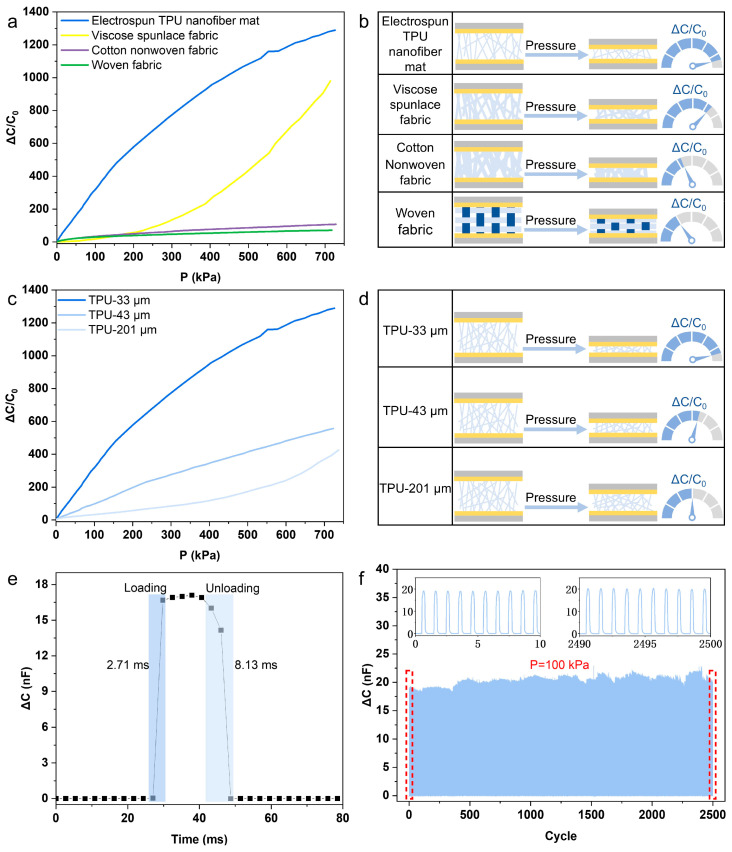
(**a**) Response-pressure curves of sensors with different ionic layer. (**b**) Schematics of working principle of pressure sensors with different ionic layer. (**c**) Response-pressure curves of sensors fabricated with TPU/IL with different thicknesses. (**d**) Schematics of working principle of pressure sensors fabricated with TPU/IL with different thicknesses. (**e**) Response curve of pressure to 80 kPa. (**f**) Durability test of pressure sensor under 2500 loading/unloading cycles.

According to the above discussion, the significant differences in microstructure (e.g., fiber diameter, arrangement, porosity, thickness) and IL loading content of four fiber-based ionic layers would result in much difference in their sensing performance. We evaluated this by comparing the sensitivity of pressure sensors fabricated from four different fiber-based materials. As shown in the sensitivity-pressure curves (Figure 3a), the electrospun TPU nanofiber-based pressure sensor demonstrated the highest sensitivity across pressures. This superior performance is directly attributed to its highest porosity, which facilitates greater IL adsorption, and its smallest fiber diameter, which creates a pronounced surface microstructure that enables a larger change in contact area during compression. A notable observation was that the viscose spunlace fabric, despite its significantly higher IL adsorption than the woven fabric and cotton nonwoven, exhibited slightly lower sensitivity in the low-pressure regime. We attribute this to its large-sized, regular semi-circular surface structure. Under low pressure, only these surface bumps compress, resulting in limited overall deformation of the ionic layer and a negligible change in the contact area. At higher pressures, the layer compresses effectively, achieving full contact with the electrodes. This increases the number of EDLs formed, and, consequently, the sensitivity rises.

The working mechanism of each sensor, correlated with material characterization results, is schematically summarized in Figure 3b. The woven fabric/IL layer exhibits low porosity and IL adsorption due to its tightly interwoven structure. Furthermore, its large-size yarns undergo minimal change in contact area under pressure, resulting in low sensitivity. In contrast, progressing from cotton nonwoven to viscose spunlace fabric and finally to TPU nanofiber mats, the fiber diameter gradually decreases while the porosity increases. This progression enhances both IL adsorption and, crucially, the magnitude of contact area change under applied pressure, leading to a corresponding increase in sensitivity.

We also investigated the influence of the thickness of the ionic layer on sensor performance. Using electrospun TPU nanofiber mat/IL of three thicknesses (33 μm, 43 μm, and 201 μm), we found that the sensor sensitivity decreases with increasing thickness (Figure 3c). The sensor fabricated with TPU nanofiber mat/IL with a thickness of 33 μm achieved sensitivities of 3.10 kPa^−1^, 1.85 kPa^−1^, and 1.02 kPa^−1^ in the pressure range of 0–200 kPa, 200–400 kPa, and 400–700 kPa, respectively. This relationship occurs because a thinner layer has lower flexural rigidity [54], allowing for greater compressive deformation under the same pressure. This leads to a more significant increase in the electrode contact area and, consequently, higher sensitivity (Figure 3d). To further validate the notion that thinner substrates and higher porosity can reduce flexural stiffness and enhance variations in contact area, we compared the compressive stress–strain curves of TPU fiber membranes with different thicknesses (33 μm, 43 μm, and 201 μm) (Figure S8a). The compressive modulus decreases significantly with decreasing thickness. The 33-μm film exhibits the lowest modulus, followed by the 43-μm film, while the 201-μm film is the stiffest. This implies that the low compressive modulus resulting from smaller thicknesses makes the material more prone to bending deformation under pressure. Additionally, we compared the compressive stress–strain curves of different substrates (Figure S8b). Under identical strain conditions, both the compressive modulus and stress values of the TPU fiber membrane were substantially lower than those of the textile material. Despite the textile’s porous nature, its response exhibited greater rigidity compared to the TPU fiber due to the stiffness of the fiber layers and their more tightly packed arrangement. At the same time, to evaluate material repeatability and inter-device variability, we verified the reproducibility of multiple batches of sensors using TPU-33 μm as the dielectric layer (Figure S9). Overall, the sensors exhibited low repeatability error and high reliability. Additionally, we quantified the sensitivity and linearity of sensors with different dielectric layers (Tables S1 and S2). Each sample exhibited distinct performance characteristics within its corresponding pressure range, enabling a systematic evaluation of sensitivity trends and linear relationships across different materials and thicknesses.

To determine the ion transport characteristics of the electric double layer (EDL), electrochemical characterization was performed using an electrochemical workstation. Figure S10a shows the phase/Bode plots of the sensor. At low frequencies, the EDL exhibits a full response with a pronounced capacitive effect; at high frequencies, the EDL response is suppressed, with resistive components becoming prominent. Furthermore, to validate the frequency dependence of the EDL, we tested the sensor capacitance versus frequency (Figure S10b). Capacitance rapidly decays with increasing frequency, as high-frequency AC signals limit ion migration time, thereby suppressing the EDL contribution. We also examined the impact of AC amplitude/bias dependence on the EDL (Figure S10c,d). Capacitance rises to a plateau region with increasing bias voltage, facilitating accumulation of dielectric ions; further bias increases trigger Faraday reactions, disrupting this ion accumulation. For AC amplitude, capacitance tends toward stability, indicating that above a certain threshold, the AC signal no longer significantly alters the dielectric double layer structure. These trends align with typical EDL behavior, confirming that capacitance modulation originates from EDL regulation rather than geometric changes.

To summarize, optimal sensing performance was achieved by an ionic layer featuring a nanofiber-based porous architecture and reduced thickness. Therefore, a TPU/IL nanofiber mat with a thickness of 33 μm was chosen for further performance evaluation. The dynamic response speed is a vital performance criterion for pressure sensors. We tested the response capability by applying a loading/unloading force of 80 kPa to the sensor using a vibrator, and the sensor showed a response time of 2.71 ms and a recovery time of 8.13 ms (Figure 3e). Given that the response time of human skin to external stimuli is about 30–50 ms, this iontronic pressure sensor shows significant promise for applications in robotic haptics and wearable health monitoring. Figure S11 shows the detection limits of the device. Even when subjected to forces of 4.47 Pa and 3.14 Pa, the sensor maintains its ability to respond to pressure. Furthermore, long-term cyclic stability is another crucial metric. We conducted dynamic load/unload cycle tests on the sensor under varying pressures to evaluate its dynamic sensing capability. Figure S12 displays the dynamic load/unload cycle test curves for the sensor across four pressure conditions. At identical pressures, the capacitance peak variation remains stable. As pressure increases, the capacitance peak grows proportionally with pressure. The sensor’s capacitance value recovers to its initial state after multiple cycles, demonstrating excellent stability and repeatability. We conducted 2500 repeated loading tests on the sensor at 100 kPa pressure. Result showed there was just a minimal signal change of 3.5% between the first and last cycles (Figure 3f), underscoring the device’s reliability. Additionally, we further validated the sensor’s durability under prolonged stress by subjecting it to an 80 kPa force over 10,000 cycles (Figure S13). Although the sensor capacitance exhibited a slight increase during long-term cycling, the repeatability of its dynamic response remained unaffected. Compared to recently reported capacitive pressure sensors, the device in this work demonstrates a competitive advantage, achieving a high sensitivity of 1.02 kPa^−1^ over a wide pressure range of 0–700 kPa. This performance surpasses that of most existing sensors based on the EDL sensing mechanism (Figure S14, Table S3).

### 3.3. Wearable Applications

**Figure 4:**
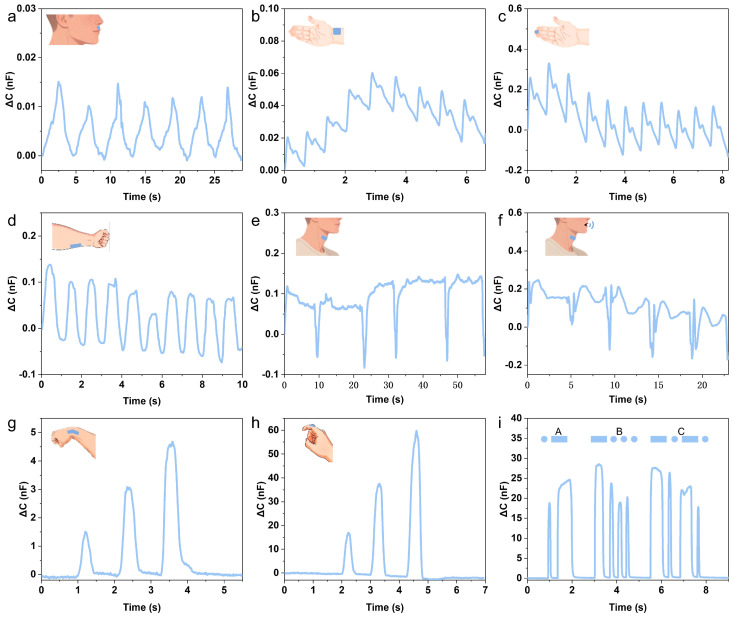
Wearable application of the iontronic pressure sensors. (**a**) Respiratory monitoring, (**b**) Wrist pulse wave detection, (**c**) Fingertip pulse wave detection, (**d**) contraction/relaxation muscle movement monitoring, (**e**) swallowing movement monitoring, (**f**) voice recognition, (**g**) wrist bending, (**h**) finger bending, (**i**) recognition of Morse Code output.

Leveraging the excellent electromechanical properties, including high sensitivity, fast response, and wide detection range, the iontronic pressure sensor was applied for diverse applications, from detecting subtle physiological signals (e.g., pulse, respiration, articulation) to measuring larger bodily motions (e.g., joint flexion, compression). To demonstrate its capability in monitoring vital signs, the sensor was first attached to a subject’s nasolabial fold using medical tape. As the subject breathed naturally, the weak pressure from the airflow induced periodic capacitance changes (Figure 4a). The respiratory rate, calculated from the peak-to-peak interval of the signal, was determined to be 15 breaths per minute. This ability to accurately monitor breathing patterns suggests the sensor’s potential for early screening of conditions such as sleep apnea, asthma, and bronchitis.

We then applied the sensor for pulse monitoring, a critical indicator of cardiovascular health that can reveal conditions like arrhythmia and arteriosclerosis. When affixed to the wrist, the sensor recorded a heart rate of 76 beats per minute (Figure 4b), with characteristic pulse wave waveforms (P wave and T wave) clearly visible [55,56]. Furthermore, the sensor’s high sensitivity enabled the detection of even weaker pulses at the fingertip, from which the corresponding characteristic waveforms were successfully extracted (Figure 4c). These results underscore the sensor’s significant potential for use in disease prevention and rehabilitation assessment. It is worth noting that the pulse signal exhibits a drift, likely due to temperature effects and motion artifacts [57,58,59]. The observed signal drift is attributable to two primary factors: (1) ion migration from the temperature sensitivity of the ionic liquid and (2) variations in pressure and contact area caused by motion artifacts and unstable mechanical contact. Both mechanisms influence the sensor’s capacitance, leading to the drift. Additionally, we have incorporated quantitative testing of sensors in wearable applications. As shown in Figure S15a, different breathing patterns yield distinct signal outputs from the sensor: deep breathing produces steady airflow, while rapid breathing accelerates air exchange frequency, reducing the interval between capacitance peaks. During breath-holding, the sensor receives no airflow-induced signals, resulting in a flatline pattern. Figure S15b,c displays pulse waveforms during different activities. Calculations indicate the test subject’s pre-exercise pulse rate was 86 beats per minute. Post-exercise, accelerated blood flow due to enhanced aerobic metabolism caused a sharp pulse increase. Monitoring confirmed the subject’s pulse rose to 114 beats per minute, validating the sensor’s efficacy in quantitative metric testing.

Then, we attached the sensor to the inner side of the forearm muscle and successfully detected the dynamic changes in capacitance during the process of clenching (muscle contraction) and relaxing (muscle relaxation), as shown in Figure 4d. When clenching, muscle contraction causes the sensor contact to weaken, resulting in a decrease in capacitance. When relaxing, the muscle restores contact and applies pressure to the sensor, causing the capacitance to increase. This functionality indicates its potential applications in the rehabilitation treatment for muscle injuries. We also recorded muscle swallowing activity (Figure 4e) and vibration phonation (Figure 4f) caused by throat movements by attaching the sensor to the throat. The resulting capacitance signals exhibited periodic changes, with phonation signals clearly distinguished between the two syllables ‘a’ and ‘p’, demonstrating the sensor’s ability to detect minute physiological signals.

In addition, owing to its wide response range, the sensor also performs effectively under high-pressure conditions, such as those generated by finger bending and tactile input for safety protection (Morse code). When attached to finger and wrist joints (Figure 4g,h), the sensor measures capacitance variations correlated with joint angle and range of motion, offering valuable data for athlete training and the guidance of proper movement techniques. It is noteworthy that throat monitoring and joint flexion produce diametrically opposed capacitive signal responses. This occurs because throat movements such as swallowing and speaking cause skin stretching and increase the electrode spacing, whereas the compressive force applied to the sensor during joint flexion reduces the dielectric layer thickness. Finally, based on the principle of Morse code, we encode information by performing short and long press operations on the sensor (Figure 4i). The output capacitance signals exhibited distinct temporal characteristics and high stability, demonstrating their potential applications in information security and intelligent robot tactile interaction. Furthermore, considering the temperature sensitivity of IL, we envision the sensor’s multimodal detection capabilities and aim to achieve high-resolution monitoring of the entire system through deep learning reinforcement [60,61,62].

## 4. Conclusions

In this study, we conducted a comparative study into the use of various fiber materials for fabricating ionic dielectric layers to develop highly sensitive iontronic pressure sensors. Four commonly available fiber substrates—woven fabric, cotton nonwoven fabric, viscose spunlace fabric, and electrospun TPU nanofiber mat—were selected to construct the ionic layers through a straightforward ionic liquid impregnation process. The influence of the microstructure and thickness of these ionic layers on sensor performance was systematically examined. Results indicate that the electrospun TPU nanofiber mats, characterized by high porosity and minimal thickness, serve as an ideal ionic layer. Its capability for high ionic liquid absorption and a microstructural surface composed of nanoscale fibers collectively contribute to superior sensor sensitivity. The optimized pressure sensor demonstrated a sensitivity of 3.10 kPa^−1^ in the 0–200 kPa range, with retained high sensitivities of 1.85 kPa^−1^ and 1.02 kPa^−1^ in the 200–400 kPa and 400–700 kPa intervals, respectively, underscoring both its high responsiveness and broad detection range. Furthermore, the sensor exhibited outstanding dynamic performance, with a rapid loading response time of 2.71 ms and an unloading recovery time of 8.13 ms, coupled with exceptional operational stability for up to 2500 continuous compression cycles. Leveraging these attributes, the sensor proved capable of accurately capturing subtle physiological activities such as pulse, respiration, and vocal cord vibrations, as well as larger pressure stimuli including joint movements. These results highlight its significant potential for applications in wearable health monitoring and human–machine interactions. Furthermore, to gain deeper insights into the structure-property relationship, our future work will involve systematic investigations into the optimal dielectric layer thickness. This will include fabricating substrates with identical thickness but varying material compositions, laying the groundwork for subsequent targeted designs of high-performance wearable sensors.

## Data Availability

The original contributions presented in this study are included in the article/Supplementary Materials. Further inquiries can be directed to the corresponding authors.

## Supplementary Materials

The following supporting information can be downloaded at: https://www.mdpi.com/article/10.3390/s25216711/s1, Figure S1: Schematic cross-sectional illustration of the sensor structure. Figure S2: The absorbed content of IL for the different fiber substrates by simple immersing. Figure S3: FT-IR spectra of (a) woven fabric and woven fabric/IL, (b) cotton nonwoven fabric and cotton nonwoven fabric/IL, (c) viscose spunlace fabric and viscose spunlace fabric/IL. Figure S4: SEM and EDS images of the woven fabric/IL. Figure S5: SEM and EDS images of the cotton nonwoven fabric/IL. Figure S6: SEM and EDS images of the viscose spunlace fabric/IL. Figure S7: Water loss rate and water vapor transmission rate of the different fiber-based ionic dielectric layer. Figure S8: Compression stress—strain curves of (a) TPU fiber membranes of varying thicknesses and (b) different fiber materials. Figure S9: Reproducibility test of sensors based on TPU-33 μm ionic fiber membranes. Figure S10: Ion transport characteristics of the EDL. (a) Bode plot; (b) Capacitance frequency dependence; (c) AC amplitude dependence; (d) Bias voltage dependence. Figure S11: Sensing signals in response to subtle pressures as low as 3.14 Pa. Figure S12: Capacitance change in the sensor when loading and unloading periodically from 20 to 80 kPa. Figure S13: Cyclic sensing performance under the loading and unloading process under 80 kPa. Figure S14: Sensitivity performance comparison of our work with existing capacitive sensors. Figure S15: (a) Detection of different breathing patterns; (b,c) Pulse detection before exercise (b,c) after exercise. Table S1: Sensitivity and linearity of different substrate materials at different pressure ranges. Table S2: Sensitivity and linearity of TPU/IL at different thicknesses under various pressure ranges. Table S3: Comparison of device performance of our device with literature-reported ones. References [48,63,64,65,66,67,68,69,70] are cited in the Supplementary Materials.

## Abbreviations

The following abbreviations are used in this manuscript:

IL	ionic liquid
EDL	electric double layer
TPU	Thermoplastic polyurethane
