# Supplementary material for: Impact of Microstructure on Sensing Performance of Fiber-Based Iontronic Pressure Sensors: A Comparative Study
Source: Sensors (Basel). 2025 Nov 3;25(21):6711. doi: 10.3390/s25216711 (PMC12609514; doi:10.3390/s25216711)
Supplement: Supplementary file 1 [file sensors-25-06711-s001.zip › sensors-3916336-supplementary.pdf]

Supporting Information

Impact of Microstructure on Sensing Performance of Fiber-Based Ionotronic Pressure Sensors: A Comparative Study

Cheng Liu [1,†], Jiaxin Xu [1,2,†], Shiman Yang [1], Yihan Xu [1], Jianyu Wang [1], Xiaoqing Liu [1,*] Li Wang [1] and Yichun Ding [1,3,4,*]

[1] School of Physics and Materials Science, Nanchang University, Nanchang 330031, China; chengliu02@163.com (C.L.); xujiaxin@fjirsm.ac.cn (J.X.); shimanyang1@163.com (S.Y.); 5714123050@email.ncu.edu.cn (Y.X.); jywang@ncu.edu.cn (J.W.); liwang@ncu.edu.cn (L.W.)
[2] Fujian Institute of Research on the Structure of Matter, Chinese Academy of Sciences, Fuzhou 350002, China
[3] Jiangxi Provincial Key Laboratory of Photodetectors, Nanchang University, Nanchang 330031, China
[4] Research Center for Chip Design, Nanchang University, Nanchang 330031, China

† These authors contributed equally to this work
* Correspondence: liuxiaoqing@ncu.edu.cn (X.L.); yichun.ding@ncu.edu.cn (Y.D.)

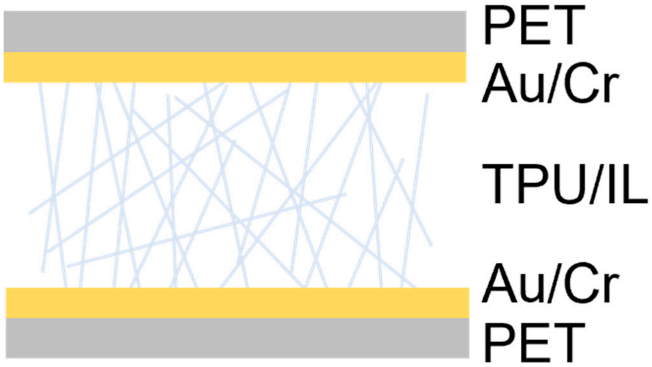

Figure S1. Schematic cross-sectional illustration of the sensor structure.

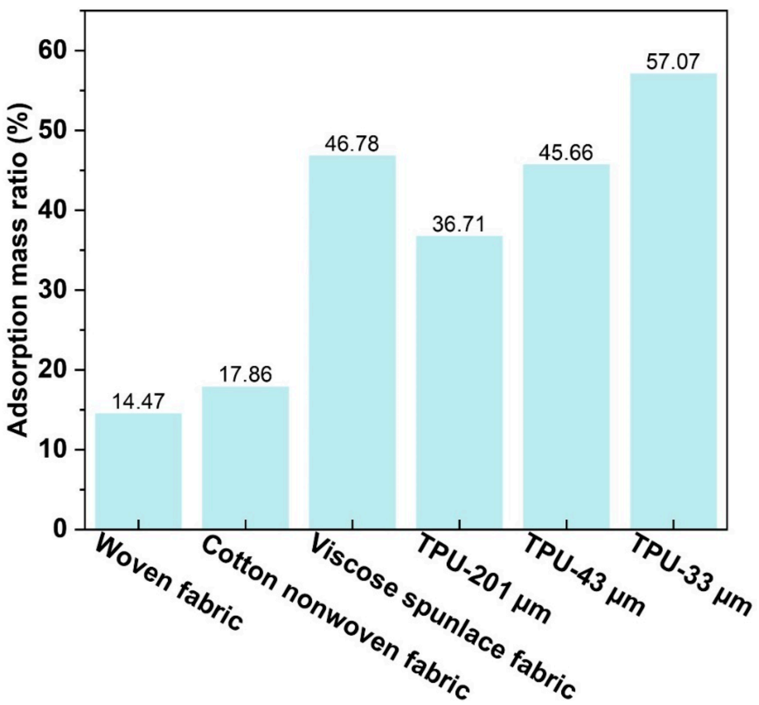

Figure S2. The absorbed content of IL for the different fiber substrates by simple immersing.

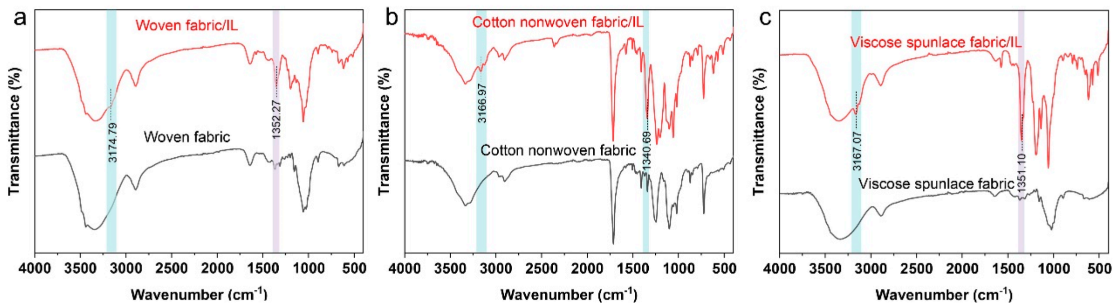

Figure S3. FT-IR spectra of (a) woven fabric and woven fabric/IL, (b) cotton nonwoven fabric and cotton nonwoven fabric/IL, (c) viscose spunlace fabric and viscose spunlace fabric/IL.

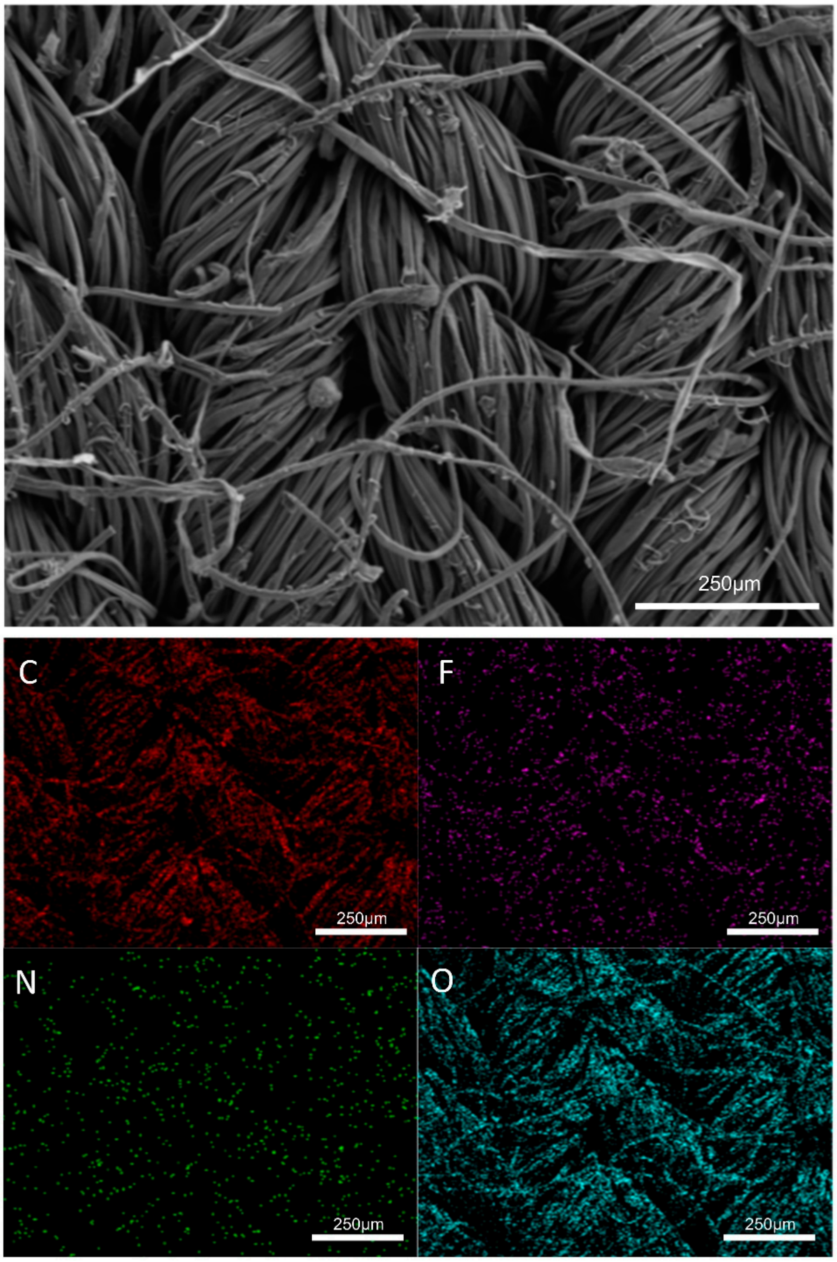

Figure S4. SEM and EDS images of the woven fabric/IL.

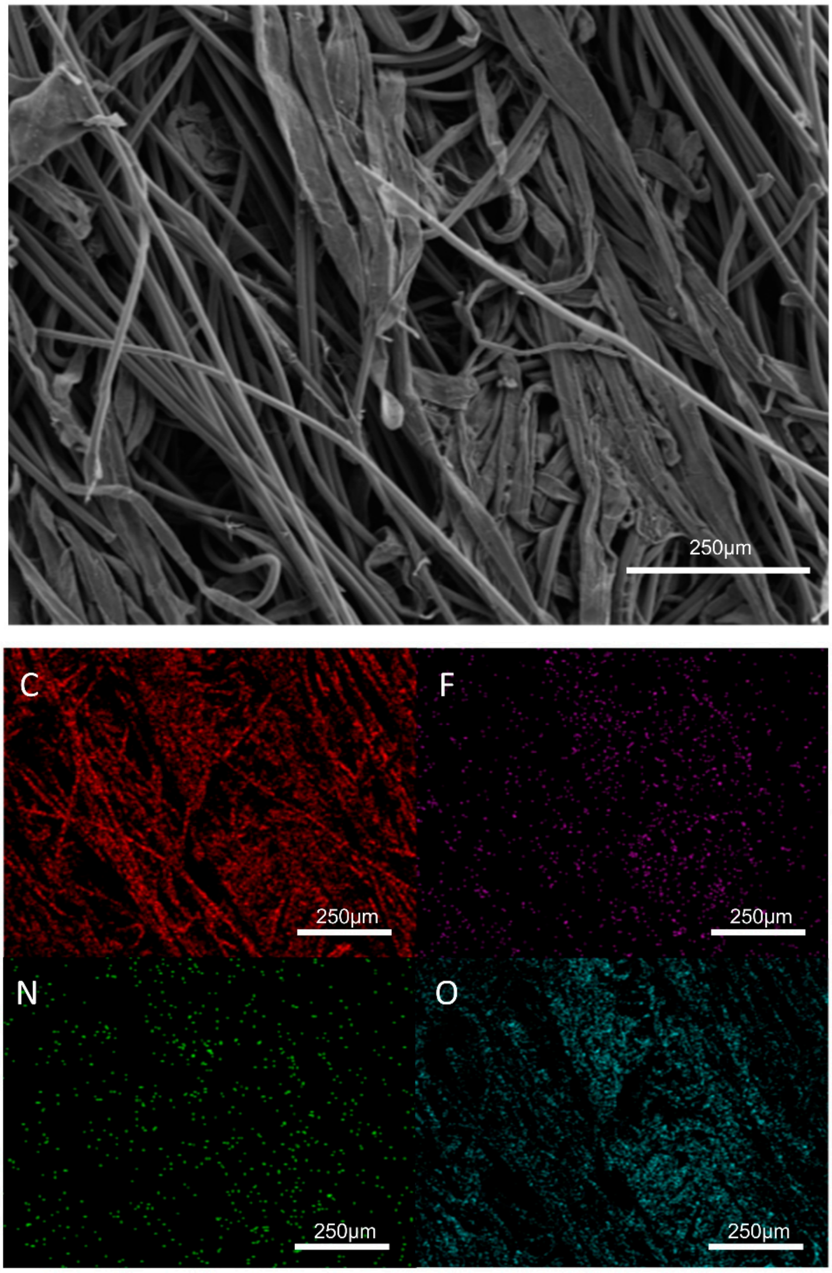

Figure S5. SEM and EDS images of the cotton nonwoven fabric/IL.

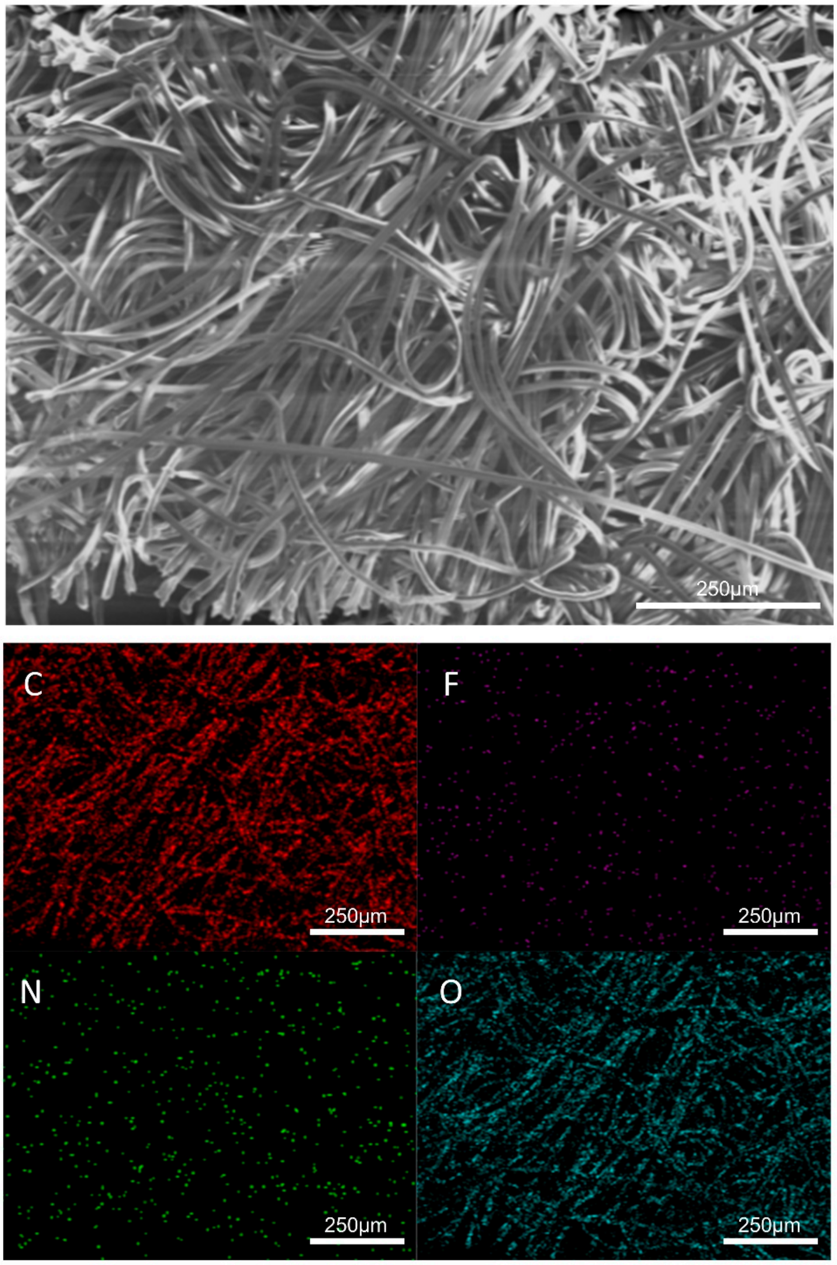

Figure S6. SEM and EDS images of the viscose spunlace fabric /IL.

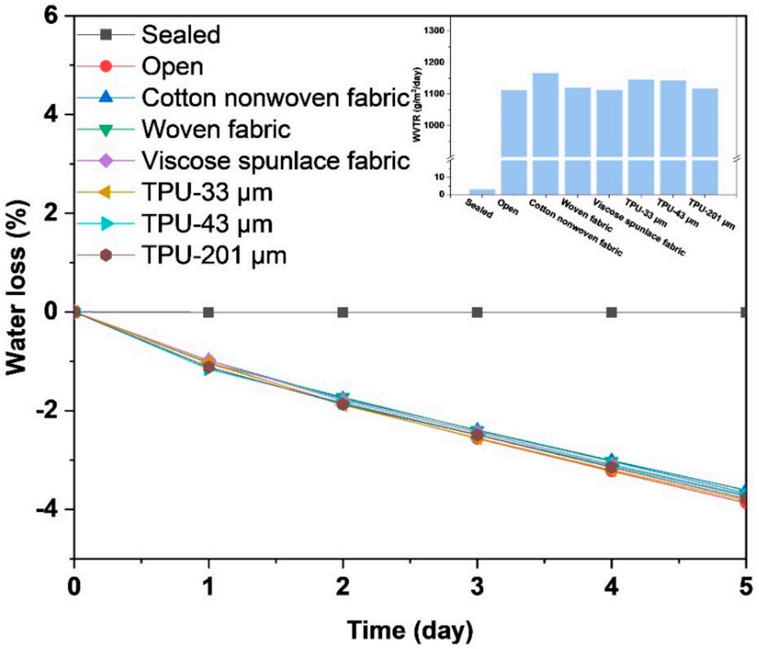

Figure S7. Water loss rate and water vapor transmission rate of the different fiber-based ionic dielectric layer.

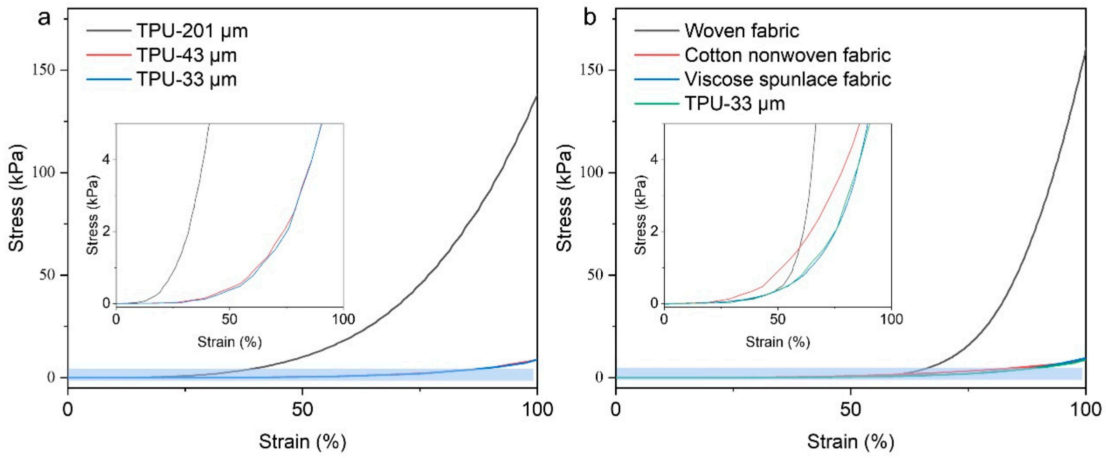

Figure S8. Compression stress-strain curves of (a) TPU fiber membranes of varying thicknesses and (b) different fiber materials.

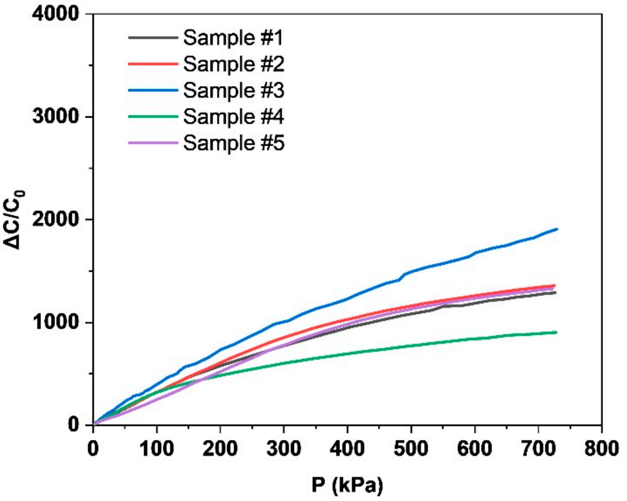

Figure S9. Reproducibility test of sensors based on TPU-33μm ionic fiber membranes.

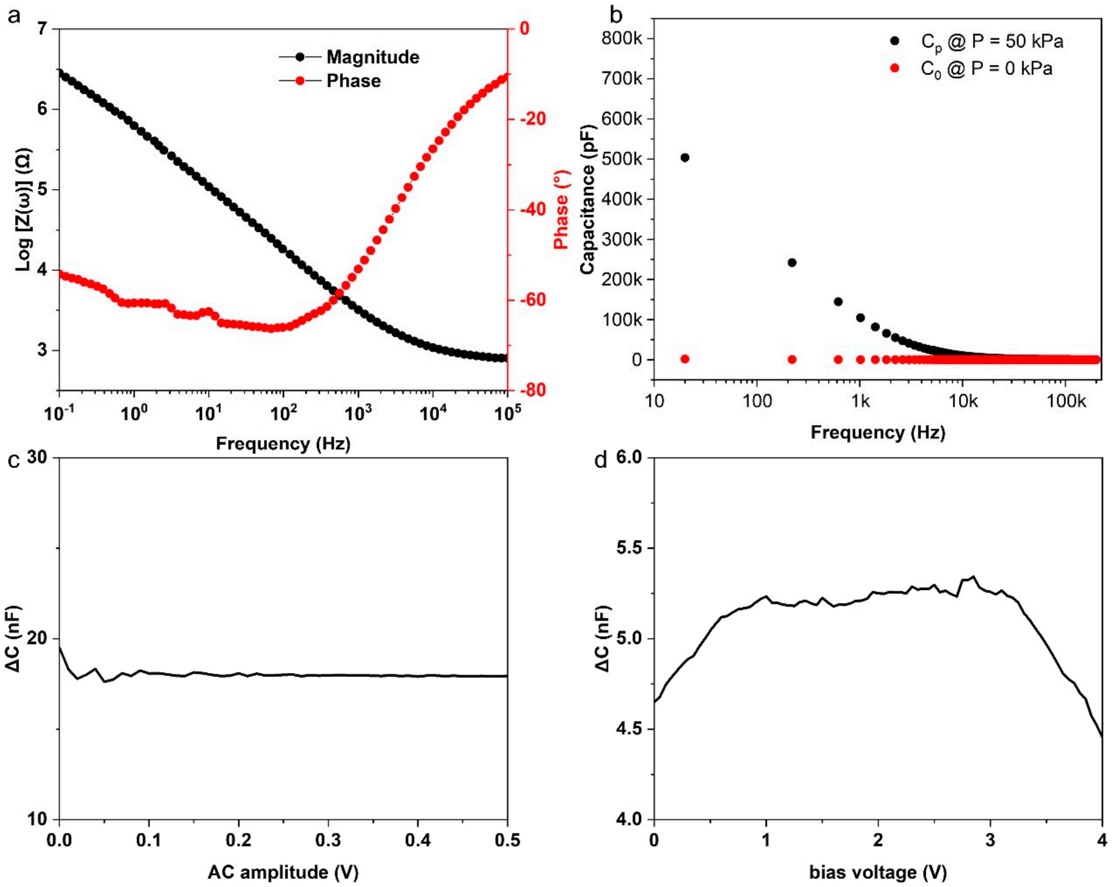

Figure S10. Ion transport characteristics of the EDL. (a) Bode plot; (b) Capacitance frequency dependence; (c) AC amplitude dependence; (d) Bias voltage dependence.

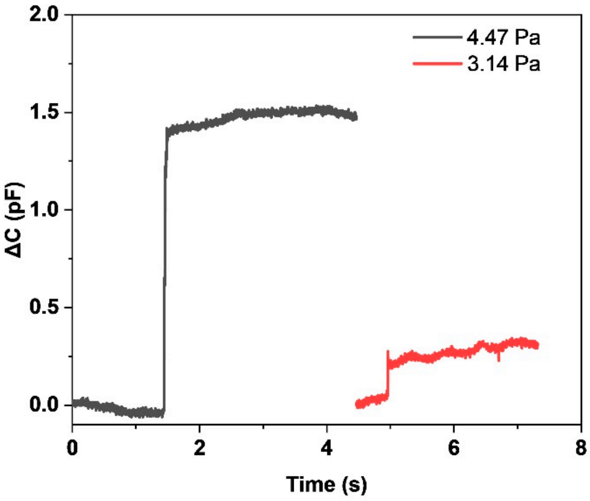

Figure S11. Sensing signals in response to subtle pressures as low as 3.14 Pa.

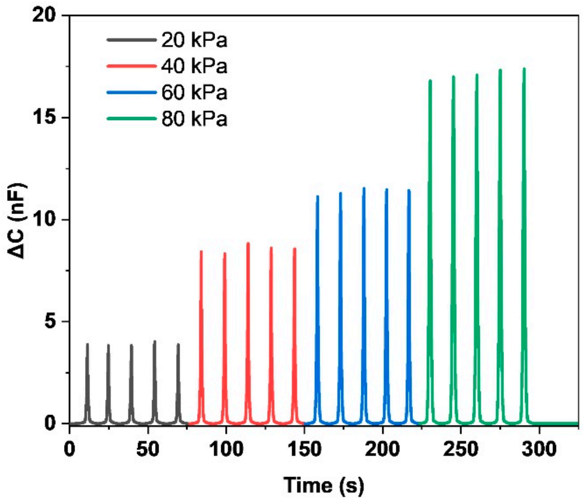

Figure S12. Capacitance change of the sensor when loading and unloading periodically from 20 to 80 kPa.

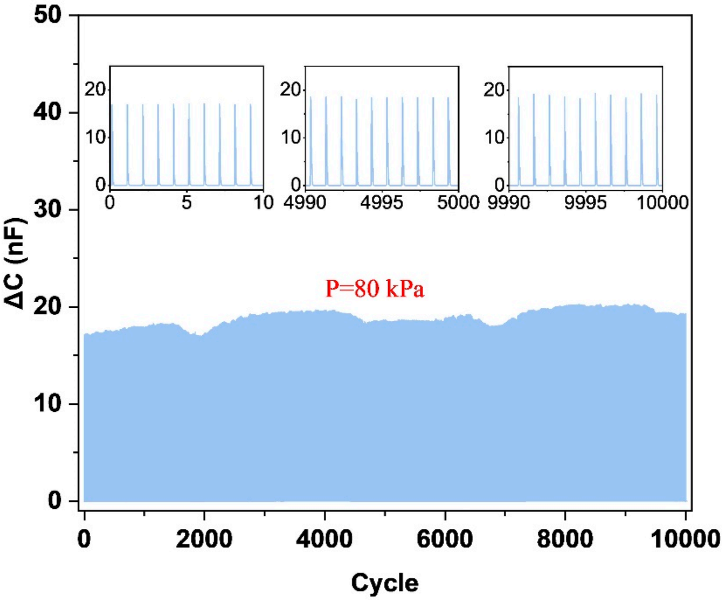

Figure S13. Cyclic sensing performance under the loading and unloading process under 80 kPa.

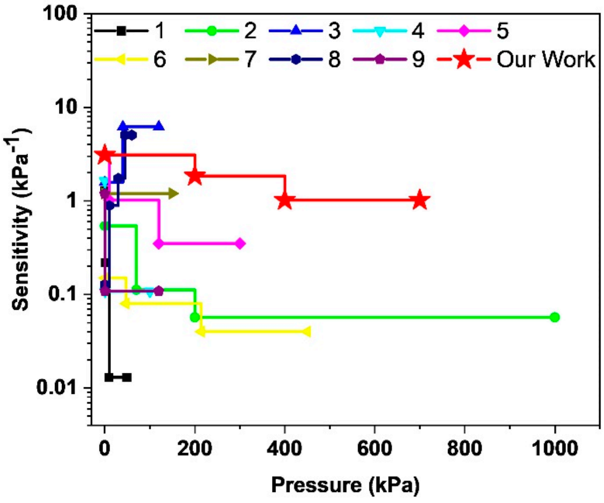

Figure S14. Sensitivity performance comparison of our work with existing capacitive sensors.

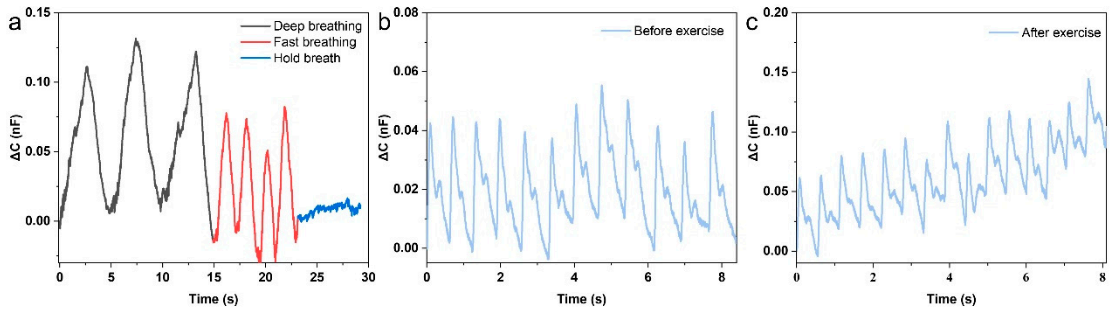

Figure S15. (a) Detection of different breathing patterns; (b-c) Pulse detection before exercise (b) and (c) after exercise.

Table S1. Sensitivity and linearity of different substrate materials at different pressure ranges

Sample	Sensitivity (kPa^{-1}), linearity (R^2)		
	Range: 0-200 kPa	Range: 200-400 kPa	Range: 400-700
Woven fabric/IL	0.20, 0.898	0.07, 0.996	0.06, 0.991
Cotton nonwoven fabric/IL	0.25, 0.954	0.14, 0.996	0.10, 0.999
Viscose spunlace fabric/IL	0.24, 0.926	0.96, 0.985	2.24, 0.993
TPU/IL-33 μm	3.10, 0.998	1.85, 0.998	1.02, 0.954

Table S2. Sensitivity and linearity of TPU/IL at different thicknesses under various pressure ranges.

Sample	Sensitivity (kPa^{-1}), linearity (R^2)		
	Range: 0-200 kPa	Range: 200-400 kPa	Range: 400-700 kPa
TPU/IL-33 μm	3.10, 0.998	1.85, 0.998	1.02, 0.954
TPU/IL-43 μm	0.96, 0.999	0.71, 0.998	0.66, 0.994
TPU/IL-201 μm	0.28, 0.984	0.29, 0.999	0.79, 0.964

Table S3. Comparison of device performance of our device with literature-reported ones.

Methodology	Microstructure	Sensitivity (kPa^{-1})	Detection Limit (Pa)	Response Time (ms)	Ref.
Electrospinning	Fiber structure	1.28 (0-0.78 kPa)<br>0.22 (0.78-9.8 kPa)<br>0.013 (9.8-49 kPa)	4.8	60/91	1
	Fiber pyramid structure	0.54 (0-70 kPa)<br>0.112 (70-200 kPa)<br>0.057 (200-1000 kPa)	1	12/16	2
	Fiber structure	1.58 (0-40 kPa)<br>6.21 (40-120 kPa)	23	170/135	3
Solution Casting	Porous film	1.64 (0-0.5 kPa)<br>0.108 (0.5-100 kPa)	1.29	93/103	4
	Semiellipsoids with micropillars	3.19 (0-10 kPa)<br>1.02 (10-120 kPa)<br>0.35 (120-300 kPa)	0.98	13/13	5
	Microcones	0.15 (0-47 kPa)<br>0.08 (47-214 kPa)<br>0.04 (214-450 kPa)	0.35	6/6	6
	Peanut-groove structure	1.2 (0-150 kPa)	0.1	2/6	7
Immersion	Porous foam	0.128 (0-10 kPa)<br>0.891 (10-30 kPa)<br>1.742 (30-45 kPa)<br>5.067 (45-60 kPa)	-	28/18	8
	Porous film	1.194 (0-0.5 kPa)<br>0.109 (0.5-120 kPa)	0.4	40/78	9
	Fiber Structure	3.1 (0-200 kPa)<br>1.85 (200-400 kPa)<br>1.02 (400-700 kPa)	3.14	2.71/8.13	Our Work

References

1. Wang, B.; Wang, J.; Lou, Y. Y.; Ding, S. S.; Jin, X.; Liu, F.; Xu, Z. J.; Ma, J. Y.; Sun, Z. M.; Li, X. Y., Halloysite Nanotubes Strengthened Electrospinning Composite Nanofiber Membrane for On-Skin Flexible Pressure Sensor with High Sensitivity, Good Breathability, and Round-The-Clock Antibacterial Activity. *Applied Clay Science* **2022,** 228, 106650.
2. Wang, X.; Li, Y.; Wang, Y.; Huang, W. C.; Zhao, X. M.; Chen, K. D.; Luo, F. C.; Qin, Y. F., Fabrication Method and Various Application Scenarios of Flexible Capacitive Pressure Sensor Based on Direct Formation of Conical Structure. *Chemical Engineering Journal* **2024,** 496, 153957.
3. Cui, X. H.; Chen, J. W.; Wu, W.; Liu, Y.; Li, H. D.; Xu, Z. G.; Zhu, Y. T., Flexible and Breathable All-Nanofiber Iontronic Pressure Sensors with Ultraviolet Shielding and Antibacterial Performances for Wearable Electronics. *Nano Energy* **2022,** 95, 107022.
4. AdigÜZel, S. P.; Ercan, N., Development Of PVDF-HFP Based Flexible Hybrid Iontronic Film for Wearable Capacitive Sensor And E-Skin Applications. *Advanced Materials Technologies* **2025,** 10, (15), 70051.
5. Zhang, X. H.; Lu, Q. X.; Zhou, L.; Zhang, W.; Zhang, X. H.; Hu, F. R., Flexible Wearable Iontronic Pressure Sensors Based on An Array of Semiellipsoids with Micropillars for Health and Motion Monitoring. *ACS Applied Electronic Materials* **2025,** 7, (5), 1820-1828.
6. Zhang, Y.; Yang, J. L.; Hou, X. Y.; Li, G.; Wang, L.; Bai, N. N.; Cai, M. K.; Zhao, L. Y.; Wang, Y.; Zhang, J. M.; Chen, K.; Wu, X.; Yang, C. H.; Dai, Y.; Zhang, Z. Y.; Guo, C. F., Highly Stable Flexible Pressure Sensors with A Quasi-Homogeneous Composition and Interlinked Interfaces. *Nature Communications* **2022,** 13, (1), 1317.
7. Huang, Y.; Zhao, L. Y.; Cai, M. K.; Zhu, J. Q.; Wang, L.; Chen, X. X.; Zeng, Y. M.; Zhang, L. Q.; Shi, J. D.; Guo, C. F., Arteriosclerosis Assessment Based on Single-Point Fingertip Pulse Monitoring Using a Wearable Iontronic Sensor. *Advanced Healthcare Materials* **2023,** 12, (29), 2301838.
8. Cetin, O.; Cicek, M. O.; Cugunlular, M.; Bolukbasi, T.; Khan, Y.; Unalan, H. E., Mxene-Deposited Melamine Foam-Based Iontronic Pressure Sensors for Wearable Electronics and Smart Numpads. *Small* **2024,** 20, (45), 2403202.
9. Liu, Q. X.; Liu, Z. G.; Li, C. G.; Xie, K. W.; Zhu, P.; Shao, B. Q.; Zhang, J. M.; Yang, J. L.; Zhang, J.; Wang, Q.; Guo, C. F., Highly Transparent and Flexible Iontronic Pressure Sensors Based on An Opaque to Transparent Transition. *Advanced Science* **2020,** 7, (10), 2000348.
